# FGF-Mediated Axon Guidance: Role of Downstream Signaling Pathways in Cytoskeletal Control

**DOI:** 10.3390/cells14110777

**Published:** 2025-05-25

**Authors:** Jiyuan Li, Hanqi Gao, Fang Liu

**Affiliations:** 1 Queen Mary School, Jiangxi Medical College, Nanchang University, Nanchang 330031, China; jiyuan.li@se21.qmul.ac.uk (J.L.); hanqi.gao@se22.qmul.ac.uk (H.G.); 2 Medical Experimental Teaching Center, School of Basic Medical Sciences, Jiangxi Medical College, Nanchang University, Nanchang 330031, China; 3 Department of Cell Biology, School of Basic Medical Sciences, Jiangxi Medical College, Nanchang University, Nanchang 330031, China

**Keywords:** axon guidance, fibroblast growth factors, FGF signaling, cytoskeleton

## Abstract

Axon guidance, a fundamental process in neural circuit formation, is intricately regulated by Fibroblast Growth Factors (FGFs) and their receptors (FGFRs) through dynamic cytoskeletal remodeling. FGF signaling, mediated by heparan sulfate proteoglycans or Klotho co-factors, activates key downstream pathways: PI3K-Akt, JAK-STAT, PLCγ, and RAS-MAPK. These pathways orchestrate actin filament dynamics, microtubule stability, and the organization of intermediate filaments. These pathways converge on Rho GTPases, cofilin, profilin, and tau to balance the cytoskeletal assembly−disassembly cycles, enabling growth cone navigation. Unresolved questions, such as the mechanisms underlying FGF-mediated growth cone steering, highlight critical future research directions. This review integrates structural, molecular, and functional insights into how FGF-FGFR interactions regulate axon pathfinding, emphasizing the crosstalk between signaling cascades and cytoskeletal plasticity. Elucidating these mechanisms not only advances our understanding of neural development but also opens therapeutic avenues for neuro-developmental disorders, nerve injury, and neurodegenerative diseases by targeting FGF-driven cytoskeletal dynamics.

## 1. Introduction

Axon guidance is a fundamental process in neural development that enables growing axons to navigate complex extracellular environments and establish precise synaptic connections. This intricate process relies on the integration of extracellular guidance cues and intracellular signaling networks that orchestrate cytoskeletal dynamics. Major conserved families of classical axonal guidance cues exist: Slits, ephrins, semaphorins, and netrins [1]. Nonetheless, many studies have demonstrated that not only classical axonal guidance cues have the effect of axonal guidance, but morphogens also induce the steering of the growth cone. Morphogen families have been classified into fibroblast growth factors (FGFs), hedgehogs (Hhs), bone morphogenetic proteins (BMPs), and Wnts [2]. Among these cues, FGFs and their receptors (FGFRs) have emerged as critical regulators of axon pathfinding. FGF-FGFR interaction modulates cytoskeletal components, including actin filaments, microtubules, and intermediate filaments, through downstream signaling pathways, such as the phosphatidylinositol 3-kinase (PI3K)-protein kinase B (Akt), Janus kinase (JAK)-signal transducer of activators of transcription (STAT), phospholipase-C gamma (PLC-gamma)-protein kinase C (PKC), and renin-angiotensin system (Ras)-mitogen-activated protein kinase (MAPK) pathways, which collectively steer growth cone motility and directional persistence [3,4,5,6,7]. The cytoskeleton serves as a structural and functional scaffold for axon guidance, with actin driving protrusion/retraction cycles, microtubules stabilizing axon shafts, and intermediate filaments conferring mechanical resilience [8]. A large number of studies have also highlighted the role of asymmetric FGFR distribution in spatially polarized signaling and second messenger concentration-dependent switch on axon guidance, offering insights into how FGFs intervene in axon guidance [9,10,11].

In this review, we synthesize the current knowledge on FGF-FGFR interaction and cytoskeletal regulation, emphasizing the interplay of downstream pathways, and discuss the mechanisms of axon navigation.

## 2. The Background of FGFs Transferring Signals Mechanism

### 2.1. FGFs and the Role of FGFs in Nervous System

The Fibroblast Growth Factor (FGF) family comprises 22 members in humans, which are classified into paracrine, endocrine, and intracrine subgroups based on their signaling modes. Paracrine FGFs (e.g., FGF1, FGF2, and FGF8) and endocrine FGFs (FGF15/19, FGF21, and FGF23) bind to FGFR tyrosine kinase receptors to regulate processes such as cell proliferation, differentiation, and metabolic homeostasis [3,12,13,14,15]. The FGF1 subfamily was first identified, and according to the isoelectric point, the FGF1 subfamily is divided into acid Fibroblast Growth Factor (aFGF; also called FGF1) and basic Fibroblast Growth Factor (bFGF; also called FGF2) [16]. Endocrine FGFs primarily mediate metabolic regulation but may indirectly influence the neuronal environment. Intracellular FGFs (FGF11–14) act independently of FGFRs and are excluded from this review, as we focus on FGFR-dependent signaling in axon guidance [3,17].

**Figure 1:**
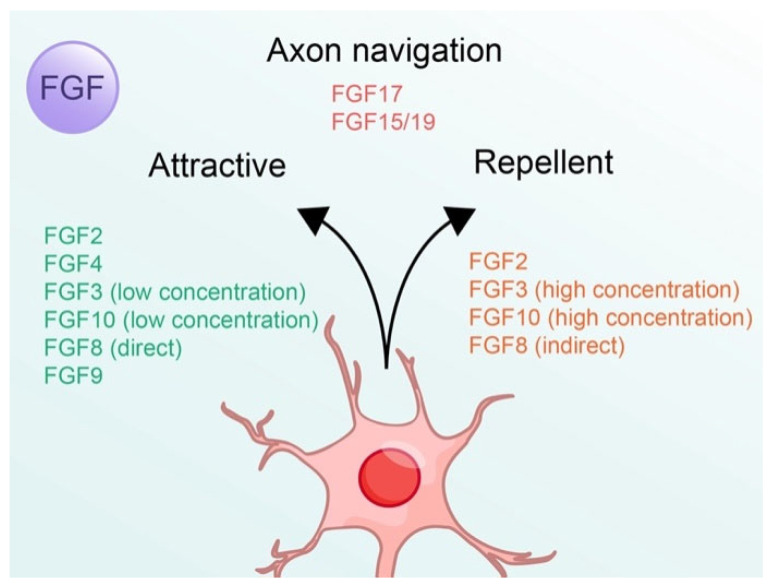
Schematic representation of FGFs that affect axon guidance. In the presence of FGF on one side, neurites deviate from their original growth direction. FGF4, 9 exerts attractive effects on axon guidance. FGF2 performs differently on different neurons, with an attractive effect on medial motor column axons and a repellent effect on retinal ganglion axons. The roles of FGF3 and FGF10 are dependent on their concentration, with attractive effects at low concentrations and repellent effects at high concentrations. FGF8 directly has an attractive and indirect repellent role in guiding axon navigation. FGF17 and FGF15/19 also affect axon guidance, but the turning direction has not been clarified. FGF: fibroblast growth factor. Green text: FGFs shown in green are designated as attractive factors in axon guidance; Red text: FGFs shown in red have the effect on axon navigation; Orange text: FGFs shown in orange are repellent factors in axon guidance.

Numerous studies have demonstrated that different FGFs have different effects on the nervous system (Table 1). However, limited research on FGF-mediated axon guidance leaves the mystery of many FGF effects on guiding axon growth cone turning (Figure 1). In the FGF1 subfamily, only FGF2 has been confirmed to mediate axon attraction and repulsion in different types of axons [18,19,20,21], whereas FGF1 contributes to axon regeneration and increases the number of axons [22]. Similarly, FGF4 exerts an attractive effect on medial motor column (MMCm) axons [18,19], while FGF6 inhibits axon regeneration in peripheral nerves [23]. The other member of the FGF4 subfamily, FGF5, has recently been discovered to play a regulatory role in granule neuron precursor expansion [24]. Interestingly, in the FGF7 subfamily, FGF3 and FGF10 have a dual effect on axon trajectory in the thalamocortical system and hypothalamus, with an attractive effect at low concentrations and a repulsive effect at high concentrations [25,26,27]. FGF7, 10, and 22 have an influence on synapse differentiation in the mammalian brain [28]. In the FGF8 subfamily, FGF8 displays direct attraction and indirect repulsion in trochlear motor axons and midbrain dopaminergic neuron axons, respectively [29,30]. In addition, a recent study reported that FGF17 has an indirect effect on axon navigation in the mammalian forebrain [31]. There are no studies on FGF18 and axon guidance, but there are emerging studies on increasing the number of axons and neuron maturation [32,33]. In addition, FGF16 from the FGF9 subfamily also affects astrocyte maturation [33]. The other two members of the FGF9 subfamily, FGF9 and FGF20, exert attractive axon guidance in MMCm axons and axon regeneration by regulating microtubules during spinal cord injury cues, respectively [18,19,34]. As for endocrine FGFs, the study indicates FGF15/19 showed axon navigation during zebrafish lens and retina development [35]. FGF21 mediates axon outgrowth via the cell adhesion molecule L1 [36], whereas FGF23 has an influence on axonal loss in the frontal lobe [37].

### 2.2. Fibroblast Growth Factor Receptors (FGFRs)

Classical FGFRs (FGFR1–4) are transmembrane tyrosine kinase receptors that bind FGF ligands extracellularly to regulate intracellular signaling [3,38]. Their extracellular region contains three immunoglobulin-like domains (DI–DIII), with DII and DIII mediating the ligand specificity. A critical auto-inhibitory “acid box” between DI and DII prevents aberrant signaling in the absence of ligands. Ligand binding induces receptor dimerization, facilitated by the transmembrane and juxtamembrane domains, which activates the intracellular tyrosine kinase domain (TKD) to initiate downstream cascades [3,39]. Alternative splicing in FGFR1–3 generates epithelial (IIIb) or mesenchymal (IIIc) isoforms with distinct ligand-binding properties [40,41], whereas FGFR4 exclusively expresses the IIIc isovent [40].

Atypical FGFR5 (FGFRL1) lacks a functional TKD and instead features a short histidine-rich intracellular tail. Although structurally homologous to classical FGFRs, its inability to directly phosphorylate effectors suggests a role in modulating FGF signaling or extracellular matrix interactions [42].

### 2.3. Interaction Between FGFs and FGFRs

FGFs signaling transmission begins with the combination of FGFs and FGFRs. Different FGFs bind to their corresponding FGFR with varying affinities. Recent research has used the affinity of FGF1 to different classical FGFRs as the criterion to determine the affinity of other FGFs for classical FGFRs, as FGF1 is a universal ligand for all types of classical FGFRs [38,43]. As for FGFRL1, some studies have shown that FGF2, FGF3, FGF4, FGF8, FGF10, and FGF22 bind strongly FGFRL1 [42,44,45,46]. Nonetheless, simple binding of FGFs and FGFRs is not stable. Co-factors stabilize FGF-FGFR binding by directly interacting with both the ligands and receptors during FGFR activation. Different classifications of FGF require different co-factors to stabilize the FGF-FGFR complex. There are two main families of co-factors that facilitate the binding between FGFs and their receptor tyrosine kinases. Heparin or heparan sulfate proteoglycans (HSPG) stabilize the paracrine FGF-FGFR complex and FGF-FGFRL1 complex, while Klotho family proteins stabilize endocrine FGF-FGFR complex [42,47].

**Figure 2:**
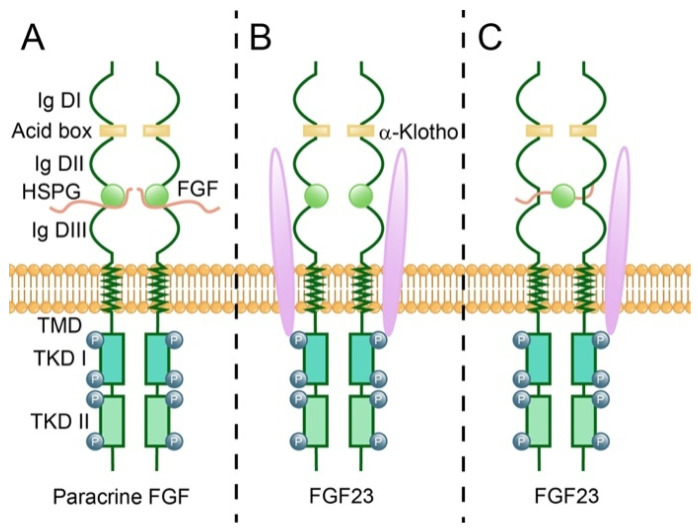
The structure of FGFRs and interaction between FGFs and FGFRs. (**A**) Paracrine FGFs interact with the corresponding FGFRs with cofactor-HSPG. The dimerization process is symmetric in the 2:2:2 FGF:FGFR:HSPG conformation. (**B**): Previous 2:2:2 symmetric conformation of the interaction between FGF23 and FGFR with α-Klotho. (**C**) Emerging 1:2:1:1 asymmetric conformation of the interaction between FGF23 and FGFR with α-Klotho and HSPG. Ig: immunoglobulin; HSPG: heparin or heparan sulfate proteoglycans; FGF: fibroblast growth factor; TMD: transmembrane domain; TKD: tyrosine kinase domain.

Heparan sulfate (HS) usually binds to cell surface proteins to form HSPG. The heparin sulfate groups (N-sulfate, 2-O-sulfate, and 6-O-sulfate) of HSPG directly interact with both FGF and their receptors (FGFR) by mechanically forming most hydrogen bonds linking the crystal structure of a dimeric FGF:FGFR:heparin ternary complex on the cell surface (Figure 2A) to facilitate and stabilize FGFR dimerization and activation [13,48,49,50]. HSPG stabilizes FGF-FGFR-heparin ternary complexes via conformational changes (e.g., D3 domain rotation) rather than forming the previously proposed crystallization-dependent canyon-like cleft between the FGFR D2 domains [13,51,52,53]. Interestingly, in addition to serving as co-receptors for receptor tyrosine kinase activation, some research has examined that heparin might also function directly as a receptor for FGFs or an activator of FGFR. For example, FGF2 binds to HSPG, inducing ERK1/2 activation, and functional analogs of heparin can activate FGFR-4 [54,55]. In addition, the FGF signaling pathway also affects heparin production, such as impaired FGFR1 signaling, inducing EXT1 expression, and decreasing HS synthesis [56,57]. Studies on the different specificities of FGF12-FGFR1c2 and FGF22-FGFR1c2 for well-defined, distinct HS structures have shown that the different specificity of HS structure can selectively activate the FGF signaling pathway, which might indicate bidirectional selection between HS and FGF signaling pathways [58]. FGF8 has been verified that it may regulate the expression of Semaphorin-3F to influence the growth of midbrain dopaminergic axons through the experiment on the existence of HSPG in mammals [30].

The Klotho gene was first discovered as an ‘anti-ageing’ gene in mice, and Matsumura et al. isolated the human homologue [59,60]. Klotho gene encodes beta-glucuronidases related membrane or secreted proteins, including three subfamilies: α-Klotho (KLA), β-Klotho (KLB), and γ-Klotho (KLG) as essential co-factors of endocrine FGFs [60,61,62,63,64]. α-Klotho facilitates FGF23 activating mainly FGFR4, whilst β-Klotho facilitates FGF15/19 and FGF21 activating mainly FGFR4 and FGFR2c, respectively [3,38,61,65]. Although the mechanism of γ-Klotho function has not yet been elucidated, recent experiments have verified that γ-Klotho is correlated with resistance to antineoplastic drugs (e.g., docetaxel) and tumor growth (e.g., inhibition of apoptosis) [62,66].

For a long time, it was thought that endocrine FGFs underwent significant modifications, resulting in the loss of their affinity for heparan sulfate binding and the acquisition of a novel systemic signaling mechanism involving either α-Klotho or β-Klotho [66,67]. In 2018, Chen et al. proposed a new view that the dimerization of stabilized ternary complexes and the subsequent activation of receptors continue to rely on the interaction with heparan sulfate, which serves as an essential cofactor for paracrine FGF signaling [65]. Despite the experimental model of the FGF: FGFR: Klotho ternary complex used by Chen et al. was 1:1:1, which did not reveal the conformation of the FGF: FGFR: Klotho: HS quaternary complex after recruitment and dimerization, Chen et al. created new ideas and hypotheses of the dimerization-induced conformation for later studies. Subsequent cryo-EM structures have unveiled the existence of an identical asymmetric 1:2:1:1 quaternary assembly of FGF23–FGFR–αKlotho–HS, in which HS plays a crucial role in facilitating the recruitment of a solitary FGFR chain (designated as FGFRS to distinguish it from the ’primary’ receptor FGFRP within the ternary complex) to the preexisting 1:1:1 FGF23–FGFR–αKlotho ternary complex (Figure 2B), thereby giving rise to the eventually asymmetric 1:2:1:1 quaternary complex (Figure 2C). In addition, this asymmetric mode of FGFR dimer conformation is also applied to paracrine FGFs that exclusively rely on HS-dependent signaling [68]. These remarkable discoveries not only subvert the formerly established paradigm of the symmetric dimer 2:2:2 FGF:FGFR:cofactor ternary complex dimer-induced conformation but also confirm that dimer FGFRs of endocrine FGFs require HS.

## 3. Effects of Cytoskeleton on Axon Guidance

**Figure 3:**
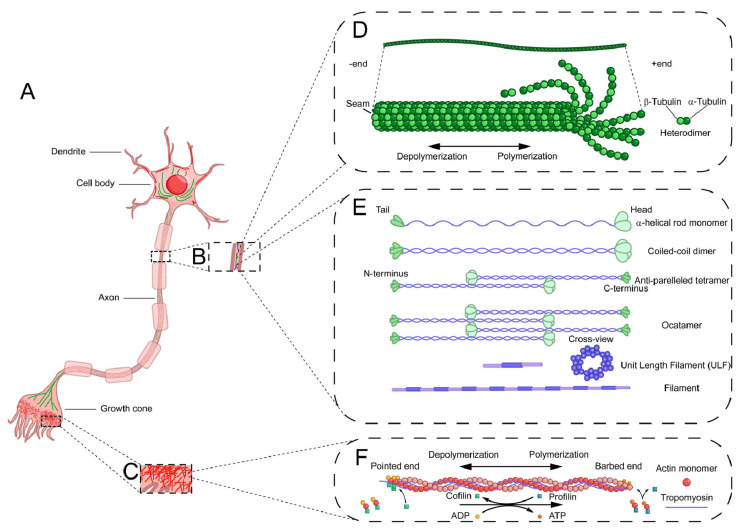
Structure of the cytoskeleton in neurons. (**A**) The distribution of cytoskeleton in neurons. (**B**) Enlarged view of the neuron axon. (**C**) Enlarged view of the growth cone. (**D**) The structure and dynamics of microtubules. (**E**) The structure and assembly of intermediate filaments. (**F**) The structure and the dynamics of the microfilament.

The cytoskeleton in eukaryotes is a dynamic, interconnected network of protein filaments comprising three cytoskeletal polymers: microfilaments, microtubules, and intermediate filaments (Figure 3D–F). These cytoskeletal polymers are essential for cellular architectural scaffolds and activities under the control of signals, such as cell migration, division, organelle trafficking, and stress adaptation [69]. In neurons, the asymmetric distribution of cytoskeleton-associated regulatory proteins induces axon guidance by precisely regulating cytoskeleton dynamics (Figure 3A) [9]. Given these studies, the cytoskeleton is indispensable for neuronal development, particularly in axonal guidance, the process by which growing axons navigate to synaptic targets. During neuronal development, growth cones (motile tips of axons) sense extracellular guidance cues and translate them into cytoskeletal rearrangement. Actin filaments drive growth cone protrusion and retraction, while microtubules stabilize axon shafts and mediate directional growth. Intermediate filaments (neurofilaments in neurons) provide mechanical resistance, enhancing the cell’s ability to withstand tension [8]. Cytoskeletal dynamics underlie neuronal guidance and play an important role in the explanation of neuron growth trajectories.

### 3.1. Microfilaments

Axon guidance, a fundamental process in nervous system development, relies on the precise regulation of growth cone motility. Growth cones, located at the axon tips, are enriched in filamentous actin (F-actin) and form dynamic structures, such as lamellipodia and filopodia. These F-actin networks enable growth cones to sense and respond to guidance cues, thereby translating extracellular signals into directional axon outgrowth. The continuous remodeling of microfilaments—through assembly, disassembly, and spatial reorganization—plays a central role in steering axons toward attractive cues and away from repulsive signals. Below, we discuss the mechanisms by which microfilament dynamics govern axon guidance.

#### 3.1.1. Microfilaments Structure

Early studies using electron microscopy and X-ray diffraction detected a double-helical structure in the right-handedness of microfilaments (actin filaments) [70,71,72]. With the development of high-resolution X-ray diffraction techniques and electron cryo-microscopy, the detection consolidated the perspective of helices-polymer structure of microfilaments and secondary structures [73,74]. Antoine Jegou and Guillaume Romet-Lemonne in 2019 summarized two descriptions of microfilament structure, left-handed single helix model with a short pitch and right-handed double helix model by two strands of globular actin (G-actin) monomers intertwined, both assembling into filamentous actin (F-actin) through ATP-dependent polymerization [69,74,75]. Tropomyosin is a double α-helical coiled-coil polymer that binds to F-actin [76]. The presence of tropomyosin in F-actin as a modulator regulates the interaction between F-actin and various actin-binding proteins [76].

#### 3.1.2. Microfilaments Regulatory Proteins

A set of important regulatory proteins, including formins, Arp2/3, myosin motors, profilin, ADF/cofilin, and capping proteins, govern the dynamics of actin filaments [77,78]. Among these, capping proteins protect F-actin from actin filament turnover, whereas Arp2/3 and myosin-gelsolin promote polymerization and depolymerization of actin filaments. In contrast, formins, as key regulators, play an important role not only in actin nucleation to stabilize untwined F-actin structure [69], but also in the interaction between profilin and F-actin to ensure the elongation of filaments [79]. Capping proteins, consisting of CapZ and tropomodulin, block the addition of G-actin monomers [80]. Arp2/3 complex contributes to the regeneration and branching of F-actin [81,82]. Myosin and gelsolin cooperatively enhance the severing of F-actin [83].

Core actin-binding proteins, like profilin and cofilin, regulate the turnover of the entire strand of F-actin at the barbed end and pointed end, respectively. The barbed and pointed ends of F-actin are the sites of filament growth and shrinkage. Profilin is a nucleation-promoting protein, assisting in the addition of free ATP-binding G-actin monomers at the barbed ends of filaments [84,85]. This polymerization is a consequence of actin-formin-profilin interactions [79]. During polymerization, formins at the bared end release profilin from the G-actin monomer, completing the polymerization process of one monomer to F-actin and the cycle of profilin [79,86]. Profilins support lamellipodial protrusion, and microtubule-F-actin coupling mutations in profilin1 impair axon growth and are linked to ALS, while formins nucleate unbranched filaments in filopodia [87]. In contrast, proteins of the ADF/cofilin are crucial for the depolymerization of actin filaments. During F-actin disassembly, cofilin binds to the monomer of F-actin at the pointed end, severing the ADP-binding monomer from the filament [85,88,89]. ADF/cofilin mediates growth cone collapse in response to repellents and facilitates F-actin turnover for attractant-driven protrusion [87]. Through the contributions of profilin and cofilin, with the assistance of regulatory proteins, actin filaments can protrude and shrink as motility. In addition, profilin, as a catalyst, exchanges ADP for ATP on G-actin, providing a monomer for profilin binding [78]. This mechanism completes the assembly cycle in an orderly manner.

#### 3.1.3. Signaling Pathways of Microfilaments Regulatory Protein and Regulation of Axon Guidance

Functionally, actin filaments drive cell motility via specific signaling proteins that regulate cofilin and profilin to control actin cytoskeletal remodeling. Rho GTPases, a subfamily of the Ras superfamily, act as molecular switches cycling between GTP-bound (active) and GDP-bound (inactive) states. Key members include RhoA, Rac1, and Cdc42 [90,91,92]. Many studies have shown that Rho mediates stimulus-induced actin filament reorganization to generate cell mobility [93,94,95,96]. In 1990, Bourne H.R. elaborated that RhoA is a small GTPase of the Rho family, as a switch of the downstream effectors [91,97]. Similarly, RhoA is phosphorylated by upstream signals, converting GDP-binding RhoA into GTP-binding RhoA [97,98]. Rac drives lamellipodia formation by activating Arp2/3-mediated branched actin nucleation, while Cdc42 guides filopodia formation and axon guidance by inducing the activation of mDia [9,92].

Rho-associated coiled-coil-containing kinases (ROCKs), comprising two highly homologous isoforms, ROCK1 and ROCK2, are serine/threonine kinases that serve as critical downstream effectors of RhoA [99,100,101,102]. While ROCK1 and ROCK2 are ubiquitously expressed, their tissue-specific enrichment—ROCK1 in non-neuronal organs (e.g., spleen and liver) and ROCK2 in the brain and muscles—suggests context-dependent roles in actin dynamics [102]. These two isoforms have been reported to have functional differences in regulating the actin cytoskeleton, but the underlying mechanisms are not fully understood [95,103,104,105]. Upon GTP-binding, RhoA attaches to the Rho-binding domain of ROCK, and a conformational shift exposes the kinase domain, enabling its activation [106]. Activated ROCK phosphorylates and activates its downstream effector p-Lin-11/Isl-1/Mec-3 kinase (LIMK) 1/2, a protein kinase with two amino-terminal LIM motifs [107,108]. The activation of LIMK1/2 subsequently phosphorylates cofilin at the binding site Ser3, inhibiting cofilin’s actin-severing activity, thereby stabilizing F-actin networks and promoting filament assembly [107,109]. Negative feedback occurs via phosphatases, like Slingshot and Cathepsin D (CathD), which dephosphorylate cofilin, restoring its severing activity and balancing filament turnover [110,111]. Furthermore, other specific pathways occur in specific tissue environments. For example, LIMK 1/2 binds to the downstream actin crosslinking protein fascin-1 (expressed in most normal adult epithelia), promoting the stability of actin filaments [112].

In contrast, mammalian diaphanous-related formins (mDia) are a subclass of the formin family of actin-nucleating proteins, serving as critical downstream effectors of RhoA, Rac, and Cdc42 in regulating actin cytoskeletal dynamics [113,114]. There are three isoforms of mDia; mDia1 is activated exclusively by RhoA, whereas mDia2 and -3 are not only in the control of upstream Rho by binding the conserved region of mDia1, -2, -3 but also are regulated by Rac and Cdc42 [115]. It has been indicated that mDias exert the different roles of elongation with different formin homology domains FH1 and FH2—binding between actin complexed and profilin by FH1 domains; assistance of assemble F-actin filaments at barbed ends by FH2 domains [116,117].

RhoA activates mDia, which binds to profilin to promote ATP-G-actin monomer delivery to growing filaments at barbed ends and plays a role in F-actin conformational conversion and the release of profilin [118]. However, in the axis of the RhoA-ROCK signaling pathway, Shao J. and Diamond MI. discovered that ROCK-1 directly phosphorylates profilin at Ser-137 site [119]. In 2012, they also reported that profilin-1 can be dephosphorylated by protein phosphatase 1 (PP1) [120]. Rac GTPases phosphorylate the downstream effector WAVE Regulatory Complex (WRC), which subsequently activates the Arp2/3 for branching [121,122]. The p21-activated kinase (PAK) communicates the Racand RhoAvia Rac/PAK/LIMK/cofilin signaling pathway [123]. Besides, it has been reported that WAVE and Arp2/3 collaboratively inhibit the effect of mDia2 [124]. However, the effect of Rac GTPases on mDia3 remains unclear. A recent study revealed the interactions among Cdc42, Arp2/3, and formins. Cdc42 recruits formins (mDia3 and PMNL1) tonucleate linear filaments and activates downstream Arp2/3 by phosphorylating WAVE (stabilized by WRC) to branch from the mother filament. Interestingly, this mechanism is dynamically regulated. Arp2/3 activates (SHIP1) to inhibit Cdc42 GTPase, which acts as a negative feedback regulator, ensuring pulsatile actin dynamics instead of sustained polymerization [125].

**Figure 4:**
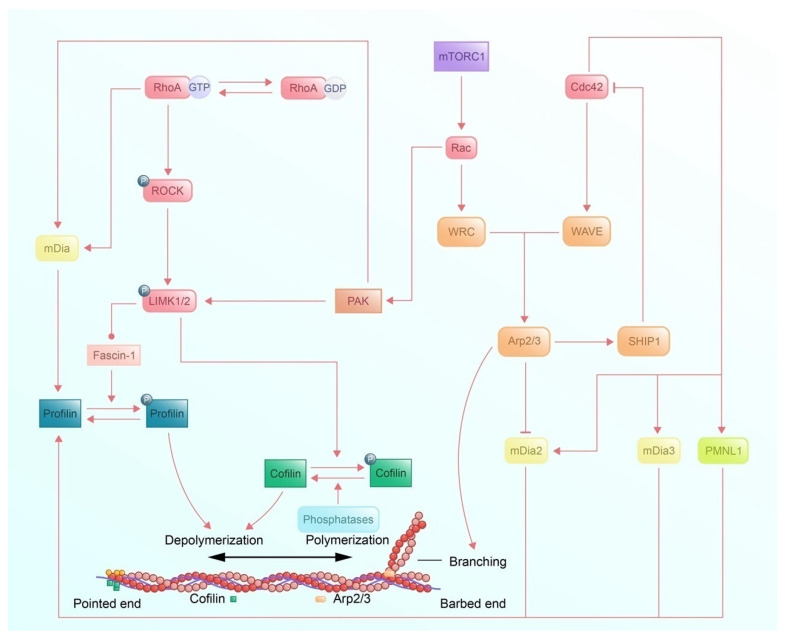
Schematic representation of the microfilament signaling pathway. Actin cytoskeletal remodeling is driven by Rho GTPases (RhoA, Rac, and Cdc42), which act as molecular switches to regulate cell motility. RhoA activates ROCK kinases, phosphorylating LIMK1/2 to inhibit cofilin’s actin-severing activity, thereby stabilizing F-actin, while phosphatases restore cofilin function for dynamic turnover. RhoA also activates mDia formins, which cooperate with profilin to deliver ATP-G-actin for linear filament elongation at the barbed ends. Conversely, Rac and Cdc42 promote branched actin networks via WRC-Arp2/3 activation, with feedback regulation that ensures pulsatile dynamics. The balance between RhoA-driven linear assembly (mDia-profilin) and Rac/Cdc42-mediated branching (Arp2/3), coupled with cofilin-phosphatase cycling, orchestrates actomyosin contractility. Red arrows: the sequence of signals activation; black arrows: the direction of actin filament dynamics.

Depending on these downstream signaling molecules, Rho GTPases mediate stimulus-induced actin filament reorganization to generate cell mobility (Figure 4). Through dual regulation, principally under RhoA GTPase control, cofilin inactivation (reducing F-actin disassembly) and profilin activation (enhancing F-actin assembly) drive actomyosin contractility and stabilize actin filaments and cell migration.

### 3.2. Microtubule

The establishment of precise neuronal connectivity relies on the microtubule (MT) cytoskeleton, which orchestrates axon guidance through mechanical support, intracellular transport, and signal transduction. During development, navigating growth cones at the axon tips integrate extracellular guidance cues to steer axons toward their targets. Emerging evidence underscores MT remodeling as a central driver of these processes, mediated by tubulin isoforms, post-translational modifications (PTMs), and MT-associated proteins (MAPs). Microtubules are not passive scaffolds but active players in axon guidance, dynamically remodeled by a network of modifiers, motors, and guidance signals. This section synthesizes the key mechanisms by which MTs regulate axon guidance.

#### 3.2.1. Microtubule Structure

MTs are dynamic, hollow cylindrical polymers composed of α- and β-tubulin heterodimers that assemble head-to-tail into linear protofilaments [126,127]. Typically, 13 protofilaments align laterally by α-α– and β-β–tubulin interactions to form a dynamic tubular structure with a slight offset instead of a closed cylinder. This slight offset causes an interface, called the *seam*, parallel to the lateral protofilament, with the interaction between α-tubulin and β-tubulin differentiated from the other protofilament contacts [128,129,130]. The number of protofilaments composing the MT is not fixed. In malaria parasites, the number of protofilaments varies from 13 to 18 [131]. This hollow cylinder has distinct polarity: a rapidly growing plus end with β-tubulin exposed and a slower-growing minus end with α-tubulin exposed, which guides the different growth kinetics of MTs [127]. Their assembly is mainly based on nucleation by the transition of γ-tubulin ring complexes (γTuRC) at non-centrosomal sites, except in neurons [132], from an open to a closed conformation within MT-organizing centers (MTOCs) [128,133].

#### 3.2.2. Dynamic Instability

The majority of MTs are in a dynamic state instead of being static, which is significant for their polymerization and depolymerization. It has been shown that the post-translational modifications, nucleotide hydrolysis, and the change of conformations driven by related regulatory proteins contribute to MT dynamics. Each dimer binds two GTP molecules: α-tubulin retains non-exchangeable GTP for structural stability and directional growth, whereas β-tubulin hydrolyzes exchangeable GTP to GDP, turning the stable GTP-tubulin lattice into an unstable GDP-tubulin lattice [134,135]. The period of MT dynamic instability begins after polymerization and ends with the GDP-locked lattice prone to depolymerization, allowing the reorganization of MTs [134,135]. Post-depolymerization, the released dimers exchange GDP for GTP at the β-tubulin E-site, restoring polymerization competence. GTP hydrolysis in β-tubulin during growth and nucleotide replacement during disassembly—underlies dynamic instability, in which GTP-binding “caps” stabilize growing MTs, whereas GDP-binding regions trigger depolymerization [136]. MT acetylation, a form of post-translational modification regulated by talin- and actomyosin-dependent mechano-sensing, integrates MT and actin crosstalk to modulate focal adhesion mechano-sensitivity, RhoA activation, and cell adhesion and migration [137,138]. Tyrosination/detyrosination cycles influence MT stability and MAP recruitment. TTL knockout mice exhibit cortico-thalamic wiring defects due to aberrant MT-actin crosstalk. Polyglutamylation licenses severing enzymes to prune MTs, ensuring motor axon targeting [139]. GTP hydrolysis and post-translational modifications in axons contribute to MT dynamic instability, in which MTs grow and shrink stochastically.

However, the precise regulation of post-translational modification—including how PTMs like acetylation or phosphorylation fine-tune the dynamics of regulatory proteins, remains incompletely understood, with conflicting evidence regarding whether PTMs stabilize or destabilize MTs. To date, many scholars have assumed three models of MT formation. The classic model of MT assembly is nucleation-elongation. Although the classic model shows the rapid formation of subunits, it cannot explain how the hollow cylinder is formed. However, one phenomenological model with multiple steps to form a structural nucleus proposed by Flyvbjerg et al. did not take into account the additional processes and was not enough to explain the hollow cylinder [140]. In contrast, one accretion model has been reported recently, which elaborates all “layer accretion” steps and describes the process of hollow cylinder formation, although experimental validation of its in vivo relevance is still lacking [136].

Growth cone turning is driven by asymmetric MT stabilization and destabilization. Netrin-1 is an attractive cue that promotes MT invasion into filopodia facing the cue. NAV1, a +TIP, crosslinks MTs and actin, thereby enhancing MT persistence in the peripheral domain. In contrast, repulsive cues, like Sema3A and Slit, induce localized MT disassembly. CRMP2, phosphorylated by the downstream kinase GSK3β, dissociates from MTs, enabling depolymerization [139]. Pioneering studies using MT-stabilizing (Taxol) or destabilizing (Nocodazole) agents demonstrated that unilateral manipulation of MT dynamics is sufficient to induce turning [141]. Similarly, asymmetric inactivation of MAPs via MICRO-CALI techniques redirects the growth cone trajectories [142,143].

#### 3.2.3. Regulatory Proteins

MT dynamics and functions are tightly regulated by a hierarchical interplay of specialized proteins, motor complexes, and destabilizing factors. End-binding proteins (EBs), core members of the MT plus-end tracking (+TIP) family, localize to growing MT tips and act as central hubs for cytoskeletal cross-talk. By recruiting adaptor proteins, such as formins (which bridge MTs to actin filaments) and cytoplasmic linker-associated proteins (CLASPs) (which stabilize MTs), EBs coordinate the spatial integration of MT-actin networks [144,145]. However, competition assays have revealed asynchronous interactions between EB-bound tubulin and actin, implicating precise spatiotemporal control over these linkages [144].

TOG domain-containing proteins further regulate MT dynamics through distinct mechanisms: *XMAP215*, essential for Ephrin-A5-induced repulsion, as a tubulin polymerase, accelerates MT elongation by promoting GTP-tubulin incorporation at the plus-ends [139,146,147], whereas *CLASPs* stabilize shrinking MTs, enhancing rescue frequency and promoting reassembly [146,147].

In parallel, kinesins and dyneins are ATP-dependent molecular motor proteins that transport cellular cargo along MTs. They also influence MT dynamics by promoting or inhibiting their polymerization. Kinesins play dual roles in MT regulation. Classified as translocators (e.g., kinesin-1, which transports cargo) or MT regulators, kinesins dynamically modulate polymerization and depolymerization. For example, kinesin-5 (Eg5) and kinesin-7 (CENP-E) enhance MT polymerization by stabilizing lattice interactions, while kinesin-8 (Kip3) and kinesin-13 (MCAK) drive depolymerization through ATPase-dependent conformational changes that destabilize tubulin subunits [148,149,150]. Dyneins (dynein-1 and dynein-2), the primary retrograde motors towards the MT minus-end, rely on dynamic MT networks for cargo transport and force generation, where +TIP proteins (e.g., EB1) and MT stability regulators (e.g., CLASPs) spatially coordinate their recruitment and activation. Dynein-2 drives retrograde intraflagellar transport (IFT) in cilia, relying on dynamic MT polymerization at the ciliary tip to coordinate bidirectional cargo trafficking with kinesin-2. While dynein processivity depends on dynein-dynactin-adaptor-cofactor (DDAC) complex-mediated anchoring to dynamic MT plus ends, its mechanism remains ambiguously tied to MT remodeling events, such as polymerization-driven EB1 gradients or catastrophe-induced tubulin turnover [138,151]. While dyneins are well-characterized in cargo transport, their direct impact on MT dynamics remains poorly understood.

MT-associated proteins (MAPs), such as MAP2, MAP4, and Tau in neurons, play pivotal roles in modulating MT initiation, elongation, and stabilization [126,152,153,154]. While all three MAPs share structural similarities—including MT-binding domains (MBDs) with poorly conserved repeat regions—their functional impacts diverge significantly. Tau induces straighter and stiffer MTs in vitro and reduces neurite branching in neuroblastoma cells, while MAP2 and MAP4, associated with dendritic structures, promote more flexible MTs and branched protrusions [155]. These differences likely stem from variations in their repeat regions, where Tau’s unique residues might enhance allosteric stiffening effects, even at substoichiometric binding. Early in vivo studies have shown that the presence of MAPs affects MT polymerization and post-translational modification of tubulin [156,157,158], and recent in vitro experiments have confirmed this MAP2 and tau [159,160]. MAP1B and Tau stabilize MTs and modulate actin interactions. Tau phosphorylation by CaMKII enables Wnt5a-mediated repulsion by reorienting MTs [139], and tau hyperphosphorylation and in vitro experiments on MAP4 polymerization effects have not been completed so far. Surprisingly, MAPs exert different functions on MTs by binding to different regions. For example, the formation of heterogenous tau-tubulin complexes can promote MT polymerization [161], whereas the binding of tau between tubulin heterodimers contributes to MT stabilization [162].

Beyond MAPs, Op18/stathmin, an MT-destabilizing protein, promotes turnover via two mechanistically resolved pathways: (1) sequestering soluble tubulin heterodimers and (2) increasing MT catastrophe rates [163,164,165]. Truncation studies have confirmed that both mechanisms operate synergistically during MT destabilization [166]. Op18/stathmin activity is physiologically suppressed by triple phosphorylation at Ser-16, Ser-25, and Ser-38, which reduces its tubulin-binding capacity and stabilizes MT networks [167]. Together, these systems—EB-mediated cytoskeletal integration, TOG-domain-driven polymerization/stabilization, kinesin-dependent mechanochemical regulation, and Op18/stathmin-controlled destabilization—establish a dynamic equilibrium that governs MT architecture, adaptability, and functional coordination with the actin networks.

#### 3.2.4. Signaling Pathways

**Figure 5:**
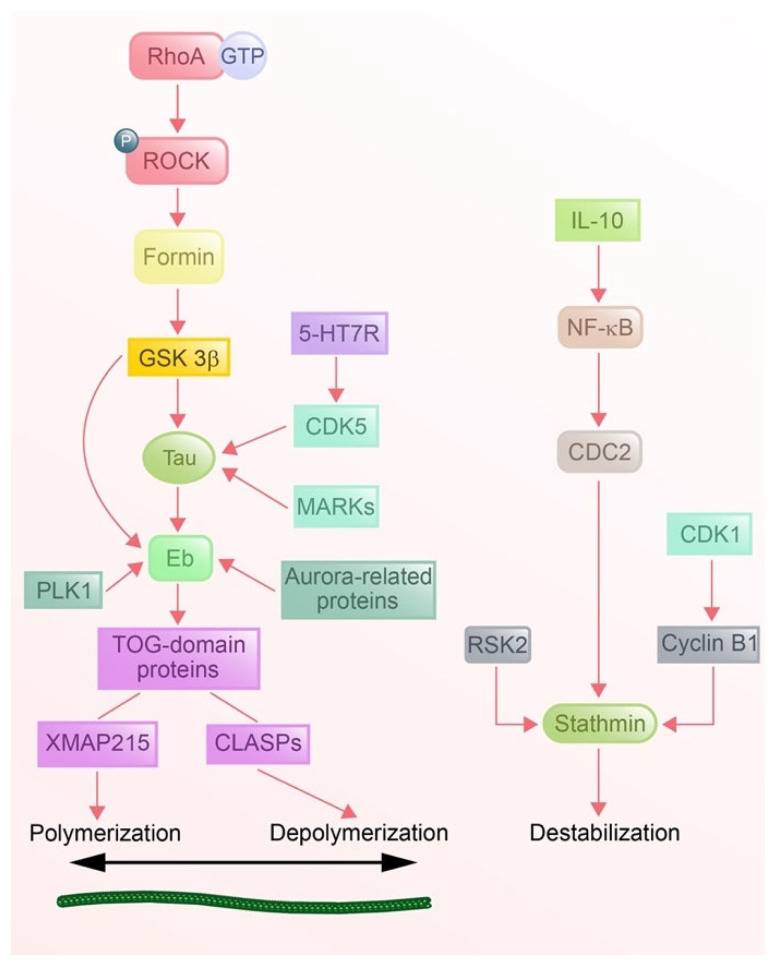
Schematic of microtubule dynamics regulated by interconnected pathways coordinating polymerization and depolymerization. RhoA-ROCK-formin-GSK3β signaling promotes polymerization via end-binding proteins (EBs), which recruit TOG-domain proteins like XMAP215, to MT plus-ends. Destabilization is mediated by stathmin (inactivated by CDK1-cyclin B1, RSK2, or IL-10-NFκB-CDC2 pathways) and balanced by stabilizing MAPs like tau, modulated by GSK3β, CDK5, and MARKs. XMAP215-EB1 interactions and tau binding ensure spatiotemporal precision. While CLASPs collaborate with EBs, their link to Rho GTPases remains unclear. Aurora kinases, Plk1, and other regulators further integrate signals, highlighting a complex network balancing dynamic MT remodeling. These signaling pathways regulate microtubules (the green tube in Figure 5) by polymerization, depolymerization or destabilization. Red arrows: the sequence of signals activation; black arrows: the direction of microtubule dynamics.

MT dynamics are tightly regulated by interconnected signaling pathways that coordinate the activities of diverse protein families (Figure 5). EBs act as central hubs, recruiting the TOG-domain protein XMAP215 to promote MT polymerization at the plus-ends under the control of the Rho GTPases-ROCK-formin-GSK3β axis in dual spatio-temporal regulation [168,169,170]. Nevertheless, there is no direct evidence to confirm that the recruitment of CLASPs is based on the Rho GTPases pathway, but the evidence on the interplay between CLASPs and EB-1 is clear [145]. Scholars have discovered more and more related proteins (e.g., aurora-related kinases) [171] and pathways (Plk1) [172] that have an effect on EBs, which might broaden the exploration of the entire MT dynamics network. Motor proteins, like kinesins and dyneins, integrate signals to orchestrate cargo transport, but the signals for MT dynamics are still not clear. Op18/stathmin, an MT destabilizer, is phosphorylated and inactivated by CDK1-cyclin B1 during mitosis [173] /IL-10-NF-κB-CDC2 axis [174]/p90 ribosomal S6 kinase 2 (RSK2) pathway [175] in response to upstream signals, enabling dynamic remodeling. Meanwhile, MT-stabilizing MAPs (such as tau, for example) are modulated by GSK3β, CDK5, and MT-affinity regulating kinases (MARKs), which phosphorylate (even hyper-phosphorylate in specific diseases) tau to regulate MT dynamics [176,177,178,179]. Proteins do not work separately and individually but in relation to each other firmly. Recently, scholars have noticed that the complicated cross-talk between pathways, such as the cooperation between XMAP215 and Eb1 with the binding of tau to Eb1 [146], ensures spatiotemporal precision in the regulation of MT dynamics.

### 3.3. Intermediate Filaments

While actin microfilaments and microtubules are well-established regulators of growth cone motility and axon guidance, the role of intermediate filaments (IFs) in these processes remains less explored. IFs), a key component of the neuronal cytoskeleton, is primarily known for providing mechanical stability and determining axon caliber. However, emerging evidence suggests that IFs also contribute to axon pathfinding by modulating structural integrity, mechanical signaling, and interactions with guidance cues.

#### 3.3.1. Intermediate Filament Structure

IFs are key components of the eukaryotic cytoskeleton. IFs have not been widely explored in biology for a long time. Increasing evidence has shown that these dynamic protein polymers are essential for cellular mechanical integrity, tissue-specific architecture, and stress adaptation [180,181,182]. IFs are composed of a diverse family of proteins with six types, including keratins, vimentin, desmin, peripheralin, glial fibrillary acidic protein (GFAP), neurofilaments (NFs), lamins, CP49/phakinin, and filen [183]. Neurofilaments, a subclass of IFs enriched in axons, contribute to the structural resilience of the growth cones. Their organization influences the mechanical properties of axons, potentially stabilizing advancing growth cones against extracellular resistance [184].

Distinguished by their intermediate diameter (~10 nm) between actin microfilaments and microtubules, IFs assemble into flexible, non-polarized helical lattices through a hierarchical multi-step assembly process [185]. IF proteins, as monomers, characterized by a central α-helical rod domain flanked by variable head and tail domains, first form parallel coiled-coil dimers via interactions between their rod domains with the N-terminus (head) and C-terminus (tail). Two dimers associate in an antiparallel, half-staggered orientation to form a tetramer, which is the basic soluble subunit of IFs. This antiparallel arrangement eliminates filament polarity, which explains the non-polarization of Ifs. Tetramers aggregate laterally into octameric protofilaments, which further assemble into unit-length filaments (ULFs) containing 16 dimers. After longitudinal annealing to form immature filaments, ULFs undergo radial compaction mediated by head-domain interactions to produce mature IFs [186,187,188]. With cryo-focused ion-beam milling, cryo-electron microscopy, and tomography, IFs assembly hierarchical multi-step mechanisms have been roughly explored in models; however, the processes that occur during IFs assembly remain incompletely understood. Critically, most assembly models are derived from in vitro reconstitution experiments, raising questions regarding their physiological relevance. Furthermore, the lack of polarity in IFs, a consequence of their antiparallel organization, might limit their role in directional processes like intracellular transport and cellular motility; however, few studies have been conducted in this field.

IFs, once considered static scaffolds, are now recognized as dynamically regulated networks that undergo continuous remodeling. IF dynamics are governed by post-translational modifications (PTMs), which modulate assembly-disassembly cycles and reorganization of IFs to adapt to various cellular functions and processes. With the development of research, more PTMs have been discovered, such as phosphorylation, ubiquitination, glycosylation, sumoylation, methylation, acetylation, and prenylation [189,190,191]. In addition, the conformation changes in the head/tail domains regulate the assembly of IFs [192,193]. On account of the lack of research on the structure of the head and tail domains, the assembly/disassembly cycle process remains unclear.

#### 3.3.2. Intermediate Filaments Regulatory Proteins

IFs are dynamically regulated by a suite of proteins, although their regulation is less understood than that of actin or microtubules. Following nerve injury, IFs, such as peripherin and α-internexin, are upregulated in regenerating axons. These IFs may enhance growth cone persistence by stabilizing nascent axonal extensions in inhibitory environments. Notably, vimentin knockout mice exhibit delayed peripheral nerve regeneration, underscoring the role of vimentin in facilitating axonal reinnervation. IFs also interact with regenerative pathways. STAT3 signaling—a driver of axon regeneration—induces vimentin expression, linking IFs to pro-regenerative transcriptional programs [184].

Some studies have reported that Cdk1, Plk1, Rho-kinase, and Aurora-B act as phosphatases to phosphorylate IFs, leading to their disassembly by disrupting subunit interactions [194,195]. However, the mechanistic diversity of PTMs across IF types (e.g., keratins vs. neurofilaments) remains enigmatic, raising questions about how tissue-specific signaling cascades tailor NF dynamics in different tissues. Phosphorylation of neurofilaments by kinases, such as Cdk5 or ROCK, can alter their assembly, indirectly affecting growth cone morphology and motility. Disruption of neurofilament networks has been shown to impair axon outgrowth in vitro, suggesting that IFs provide a scaffold that facilitates cytoskeletal coordination during navigation [184]. Sacsin, a protein translated from the SACS gene, disrupts initial assembly with a J domain (SacsJ) [196]. Studies have shown that SacsJ is a site of Hsp70 chaperones, and experiments have confirmed that Hsp70 chaperones have no effect on in vitro filament assembly [197]. However, sacsin’s selectivity for specific IFs and its role in disassembly is poorly defined. Similarly, plectin, a crosslinking protein, stabilizes IF networks to exert cellular kinematics. Recently, a new study has shown that in the absence of PTMs, the positively charged desmoplakin (DP) C-terminus binds plectin repeat 14 to recruit IFs, whereas phosphorylation/methylation reverses its charge, enabling binding to repeat 17 and repelling IFs [191]. Therefore, the regulatory effects of these proteins do not proceed unchecked, and PTMs play a key role in balancing IFs’ dynamics. Generally, despite studies on assembly and related proteins increasing, these are not enough to explain IFs dynamics mechanisms and the IFs network still remains wide blank.

## 4. Downstream Signaling Pathways

The phosphorylated dimer FGFR complex triggers different downstream intracellular signaling pathways, which have been thoroughly elucidated through extensive research. Among these, there are four key signaling cascades: the Phosphatidylinositol-4,5-bisphosphate 3-kinase/protein kinase B (PI3K-Akt), JAK-STAT, PLC-gamma, and RAS-MAPK pathways. The PI3K-AKT pathway plays a crucial role in cell growth, survival, and induced cell cycle progression, while the JAK-STAT pathway regulates cellular fibrotic processes, including proliferation, senescence, and autophagy, in response to various FGFs [198,199,200]. The PLC-gamma pathway is involved in the regulation of the cell cycle, and the RAS-MAPK pathway regulates cell proliferation, differentiation, and apoptosis [201,202]. Understanding the intricate interactions and mechanisms of these downstream signaling pathways triggered by the activated FGF-FGFR-HS complex is crucial for elucidating the biological functions and potential therapeutic targets of various diseases. In this review, we discuss the effects of the four FGF downstream signaling pathways on axon guidance.

### 4.1. PI3K-Akt Pathway

The PI3K-Akt signaling cascade, a central downstream effector of FGFR activation, plays a pivotal role in coordinating cytoskeletal dynamics to regulate axon guidance and navigation in the developing forebrain. While FGFR isoforms exhibit structural and ligand-binding heterogeneity, they converge on the PI3K-Akt signaling pathway, ensuring precise spatiotemporal control of axon pathfinding through cytoskeletal remodeling [203]. This pathway’s multifaceted roles in metabolism, survival, proliferation, and cytoskeletal organization make it indispensable for neuronal wiring [204], although its complexity raises questions about how specificity is achieved across diverse cellular contexts. A recent study has shown that axon guidance can be regulated via the PI3K-AKT-mTOR signaling pathway [205]. This pathway is intricately intertwined with numerous pathways and plays a pivotal role in governing cytoskeletal development and axon guidance in neuronal cells [206]. Elucidating the complex mechanisms underlying the PI3K-AKT signaling cascade holds significant scientific value in understanding neuronal growth and axon guidance through the regulation of cytoskeletons.

#### 4.1.1. Activation of PI3K-Akt by FGFR

FGFR dimerization and autophosphorylation recruit adaptor proteins, such as phosphorylated fibroblast growth factor receptor substrate 2α (FRS2α), growth factor receptor-bound protein 2 (GRB2), GRB2-associated binder 1 (GAB1), CRK-like proto-oncogene adaptor protein (CRKL), and phosphatases (SHP2), to form a signaling complex that activates PI3K [207]. Intriguingly, while GRB2 can bind FGFR directly, genetic studies suggest that this interaction is dispensable for embryonic development and homeostasis, implying redundancy or compensatory mechanisms in PI3K activation (e.g., SHP2 or Src-mediated pathways) [208,209]. Src family kinases further amplify PI3K-Akt signaling through tyrosine phosphatase activity, highlighting the crosstalk between FGFR and non-receptor tyrosine kinases [209,210]. Such redundancy might ensure robust pathway activation during critical developmental windows, although the relative contributions of direct and indirect PI3K activation in axon guidance remain unresolved.

#### 4.1.2. Structural and Functional Complexity of PI3K

Various types of PI3Ks are present in higher eukaryotic cells; however, class Ia PI3Ks predominantly generate D-3 phosphoinositides upon stimulation by growth factors. Class Ia PI3Ks function as heterodimeric complexes consisting of a regulatory subunit (commonly p85 with five isoforms: p85α, p85β, p50α, p55α, and p55γ, encoded by three genes, *PIK3R1*, *PIK3R2*, and *PIK3R3*) and a catalytic subunit (p110 with three isoforms: p110α, β, and δ, encoded by *PIK3CA*, *PIK3CB*, and *PIK3CD*, respectively), which together orchestrate the PI3K signaling pathway. Among the subunits, the regulatory subunit can independently act as a critical adaptor, being free in the cell. The prevailing concept is that free p85 protein exists in a dimeric form that can adopt two distinct conformations. These conformations are influenced by the interactions between the protein’s N-terminal and C-terminal domains. The monomer-dimer equilibrium of p110-independent p85 is the basis for the stabilization of fundamental pathways [211]. While the presence of free p85 is known to play a significant role in stress response pathways within the cell, isoform-specific roles are not yet completely understood, as their differential expression or activation can fine-tune cytoskeletal outputs.

#### 4.1.3. Akt Activation and Downstream Targets

PI3K, with the 3-hydroxy position of the inositol ring, catalyzes phosphatidylinositol 4,5-bisphosphate [PI(4,5)P2] to second messenger phosphatidylinositol 3,4,5-trisphosphate [PI(3,4,5)P3], a membrane lipid recruiting pleckstrin-homology (PH) domain-containing downstream signaling effectors like Akt. PIP3′s spatial distribution is tightly regulated by phosphatase and tensin homologue (PTEN), which counteracts PI3K by dephosphorylating PIP3 [212,213]. Phosphorylation of Akt is facilitated by the kinase phosphoinositide-dependent kinase 1 (PDK1), which is drawn to the PIP3-enriched sites on the membrane. Given the movement property of the lipid membrane, PIP3s separately carrying Akt and PDK1 gradually become proximate. Akt becomes fully active when two sites, threonine 308 (T308) and serine 473 (S473), are modified by the addition of phosphate groups. This process is carried out by two complexes: the cytoplasmic complex of PDK1 assembled with Smurf1 and SETDB1 (cCOMPASS) for T308, and the mechanistic target of rapamycin complex 2 (mTORC2) for S473 [214,215].

#### 4.1.4. Cytoskeletal Regulation by PI3K-Akt

**Figure 6:**
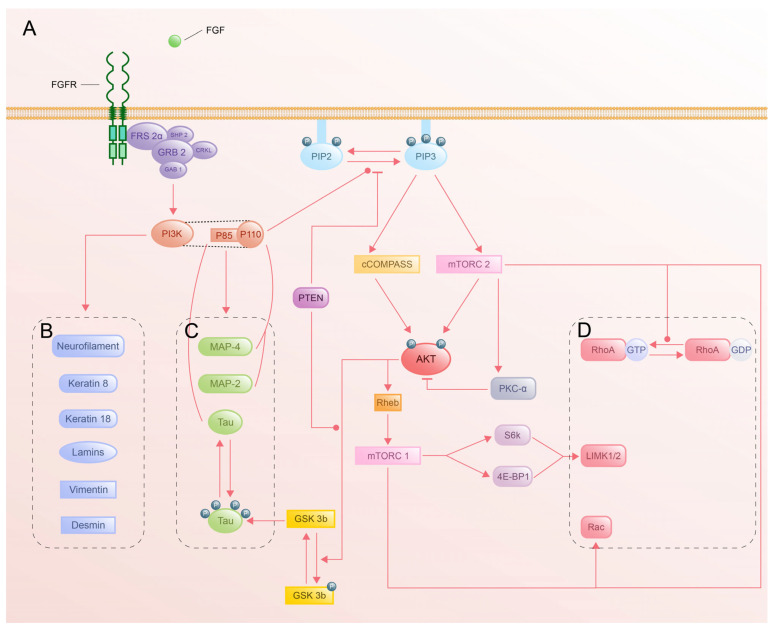
Schematic representation of PI3K-Akt pathway’s role in coordinating cytoskeletal dynamics during axon guidance by regulating microtubules, intermediate filaments, and actin networks. (**A**): PI3K activated by FGFR activation participates in phosphorylation of PIP2 to PIP3. PIP₃ recruits AKT through cCOMPASS and mTORC2. Akt’s Rheb-mTORC1-S6K1/4E-BP1-LIMK axis further modulates actin, with PKC-α providing negative feedback to limit stabilization. FGF signaling integrates these pathways and balances transient actin remodeling and microtubule-IF interactions to guide axon guidance. (**B**,**C**): PI3K activates microtubule-associated proteins (e.g., tau via p85 and MAP-4/-2 via p110), while Akt phosphorylates GSK-3β to stabilize microtubules and crosstalk with F-actin depolymerization. Akt also enhances IF synthesis (keratin 8/18 and neurofilaments) for structural support, although the neuronal IF mechanisms remain unclear. (**D**): Actin dynamics are governed by Rho GTPases: mTORC2 activates RhoA to drive linear F-actin assembly via mDia-profilin and inhibits cofilin via ROCK-LIMK, while mTORC1 derepresses Rac-Arp2/3 for branched actin nucleation, enabling growth cones to switch between exploratory and consolidatory states. Red arrows: the sequence of signals activation.

The PI3K-Akt pathway orchestrates cytoskeletal dynamics to steer axon guidance through the coordinated regulation of microtubules, intermediate filaments (IFs), and actin networks (Figure 6). While its roles in survival and growth are well-established, emerging evidence highlights its nuanced control of cytoskeletal architecture during navigation.

Activation of the PI3K-Akt pathway modulates cytoskeletal dynamics and directs axon guidance. Studies have shown that tau is activated by p85 of PI3K, while MAP-4/-2 interacts with p110 of PI3K to play a role in microtubule stability [216,217,218]. Similarly, phosphorylation of downstream Akt directly acts on GSK-3β to prevent the phosphorylation of tau, which triggers microtubule disassembly and acetylation [219]. Intriguingly, recent work reveals tau’s dual role in F-actin depolymerization, suggesting a broader function in cytoskeletal crosstalk [220]. Despite the increasing number of studies on microtubules and the PI3K-Akt pathway, many mechanisms and binding sites of other regulatory proteins of microtubules remain unclear.

Phosphorylated Akt upregulates keratin 8/18, augmenting IF abundance in epithelial contexts [221], while PI3K-Akt activation enhances neurofilament synthesis to support axon radial growth [222]. However, the mechanistic link between PI3K-Akt and IFs in neurons remains unclear. Do IFs passively reinforce the axon structure, or do they actively modulate signaling? This gap underscores the need for neuron-specific studies to dissect IF dynamics.

The effect of the PI3K-Akt pathway on actin filaments is principally based on Rho GTPase. The mechanistic target of rapamycin complex 2 (mTORC2) has been reported to participate in the conversion of RhoA GTPase from RhoA GDPase. Through RhoA-mDia-profilin/RhoA-ROCK-LIMK-cofilin pathway, mTORC2 regulates F-actin polymerization. Furthermore, mTORC2 collaborates with mTORC1 to suppress mDia2, derepressing the Rac-Arp2/3 pathway and driving branched actin nucleation. This dual regulation may enable growth cones to switch between exploratory (Arp2/3-driven) and consolidatory (mDia-driven) actin states [223]. Phosphorylated Akt plays a role in regulating actin filament dynamics via activation of the Rheb-mTORC1- ribosomal protein S6 kinase 1 (S6K1)/eIF4E-binding protein 1 (4E-BP1)-LIMK pathway [224]. PKC-α (induced by mTORC2) exerts negative feedback by inhibiting Akt phosphorylation, promoting F-actin filament depolymerization [225,226]. This negative regulation likely ensures transient actin remodeling during axon guidance, thereby preventing aberrant stabilization.

### 4.2. JAK-STAT Pathway

The interplay between growth factor signaling and cytoskeletal remodeling is pivotal for axon guidance during neural development. Emerging studies have highlighted the importance of the JAK-STAT pathway in axon guidance. For example, in *Drosophila*, JAK-STAT signaling is required for the precise targeting of photoreceptor axons in the brain, with genetic screens implicating STAT92E (a homolog of mammalian STAT5) as a key player [227]. These findings suggest that the JAK-STAT pathway is not only essential for various developmental processes but also plays a critical role in precisely wiring neuronal circuits. While the FGF system is well-known for its roles in neuronal survival, differentiation, and synaptic plasticity, its interaction with the JAK-STAT pathway to regulate cytoskeletal dynamics remains underexplored. This section synthesizes current insights into how FGF-mediated JAK-STAT signaling influences cytoskeletal components—actin filaments, microtubules, and intermediate filaments, to steer axon navigation.

#### 4.2.1. FGF Signaling and JAK-STAT Activation

FGF ligands bind to FGFRs, triggering receptor dimerization and autophosphorylation of specific tyrosine residues within the receptor’s intracellular domain, initiating the downstream JAK-STAT signaling pathway. The JAK-STAT signaling pathway is a universally expressed intracellular signal transduction pathway involved in many crucial biological processes, including cell proliferation, differentiation, apoptosis, and immune regulation [228,229]. In non-neuronal contexts, aberrant FGFR-JAK-STAT activation drives pathologies such as prostate and breast cancers. While direct evidence of the FGFR-JAK-STAT pathway acting on the cytoskeleton in neurons is limited, it has been reported that Eph upregulates JAK2-STAT1/3 activity on actin filaments to direct growth cone navigation [230]. This suggests that potential JAK-STAT signaling pathway modulating cytoskeletal dynamics in axonal pathfinding may be equally critical under the activation of FGFR. Given the lack of direct evidence of the FGFR-JAK-STAT signaling pathway on axon guidance, we focus on the activation of the JAK-STAT pathway, STAT-induced cytoskeleton dynamics, and JAK-STAT mediated axon guidance separately.

#### 4.2.2. JAK-STAT Signaling Pathway by FGFR Phosphorylation

Phosphorylated tyrosine residues serve as docking sites for various signaling molecules, including the JAK family of tyrosine kinases. The JAK family mainly comprises four members: JAK1, JAK2, JAK3, and Tyk2, with over 1000 amino acids and a molecular weight of ~130 kDa. JAK proteins, a group of non-transmembrane tyrosine kinases, particularly JAK2 in the context of FGFR signaling, are recruited to and activated by the phosphorylated FGFR. This activation involves the phosphorylation of JAK proteins, which in turn phosphorylates the receptor further. Signal Transducer and Activator of Transcription (STAT) proteins are then recruited to the phosphorylated receptor. Upon recruitment, STAT proteins are phosphorylated by JAK, which triggers their dimerization. STAT family is one of the most crucial cytokine-activated transcription factors in the cytokine response, with molecular weights ranging from 79 to 113 kDa, and is composed of seven members: STAT1, STAT2, STAT3, STAT4, STAT5A, STAT5B, and STAT6 [231,232]. Beyond their transcriptional roles, recent work highlights the non-canonical and localized functions of STATs in axons, thereby expanding their regulatory repertoire.

#### 4.2.3. Cytoskeletal Regulation via Activation of STAT

**Figure 7:**
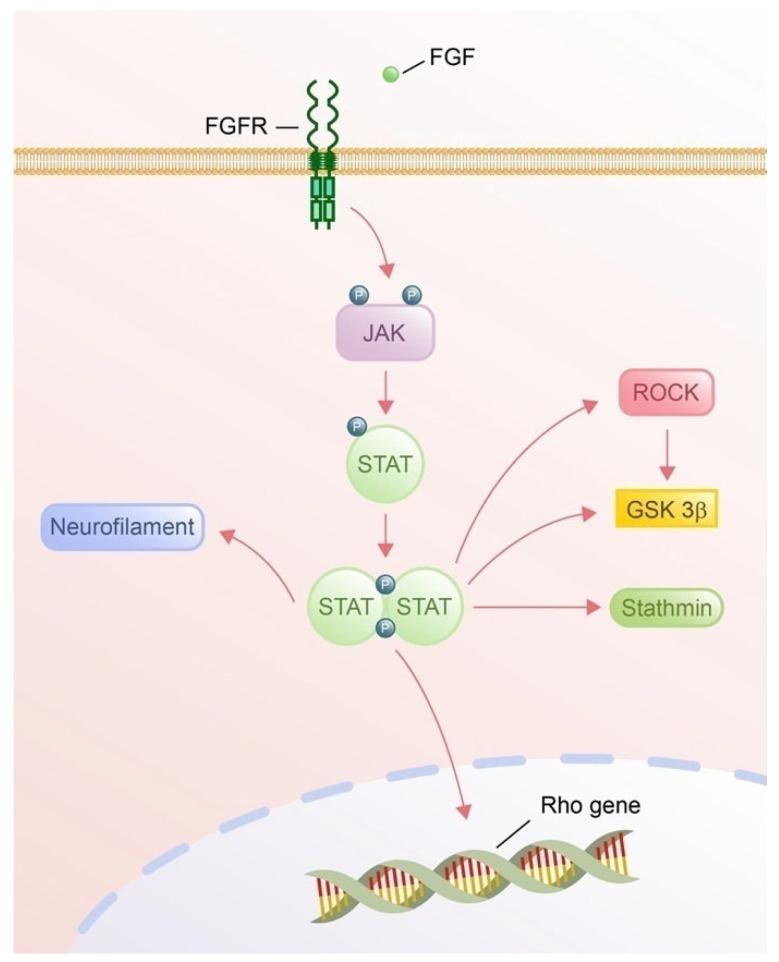
Schematic representation of the JAK-STAT pathway’s dual role in regulating cytoskeletal dynamics through transcriptional and non-transcriptional mechanisms. Activated by FGF-FGFR signaling, STAT proteins transcriptionally control Rho GTPases (e.g., via STAT6-dependent RhoA promoter activity) to influence actin remodeling and cell migration, while STAT1/3 balance Rac/Cdc42 activity. Non-transcriptionally, STAT3 interacts with stathmin in axons to stabilize microtubules, counteracted by inhibitory GSK3β and ROCKII phosphorylation post-injury. STAT signaling enhances neurofilament organization and intermediate filament dynamics, thereby supporting axonal guidance. Red arrows: the sequence of signals activation.

The JAK-STAT pathway modulates cytoskeletal components, actin filaments, microtubules, and intermediate filaments through transcriptional and non-transcriptional regulation mechanisms (Figure 7). STAT, a key transcription factor, interacts with Rho GTPase genes in the nucleus to regulate the expression of Rho GTPases. Luciferase assays and siRNA knockdown have shown that mutations in STAT6 binding sites abolish RhoA promoter activity and that STAT6 inhibitors block IL-13/TNF-α-induced RhoA upregulation, highlighting pathway dependency and proving the necessity of STAT for Rho GTPase transcriptional regulation [233]. In addition, STAT1 signaling in monocytes inhibits migration by dysregulating Rac/Cdc42 cycling, leading to aberrant actin polymerization and loss of cell polarization, while STAT3 promotes cell migration by crosstalk with Rac/Cdc42 [234]. While no direct evidence links JAK-STAT to axon guidance via Rho GTPases regulation, the regulation of Rho GTPases and actin-binding proteins (e.g., cofilin) by JAK-STAT in other cell types provides a plausible mechanistic framework. STAT1 and STAT3 may act as molecular switches to balance cytoskeletal dynamics in growth cones by integrating cytokine signals with guidance cues. Future work should prioritize neuronal models to validate these hypotheses and explore the signaling pathway of the link betweeenFGFR-JAK-STAT-mediated axon guidance.

Rather than being transported to the nucleus to activate transcription, most activated STAT3 interacts locally in axons with the microtubule-destabilizing protein stathmin, critically mediating the non-transcriptional regulation of microtubule dynamics and promoting the stabilization of microtubules [235,236]. STAT3 activation may promote pro-regenerative microtubule dynamics in axons, but its effects are counteracted by elevated GSK3βand ROCKII after central injury (DRI), which destabilize microtubules via phosphorylation of microtubule-associated proteins and actomyosin contractility, respectively, impairing axon growth capacity [237]. This imbalance between STAT3-mediated regeneration signals and inhibitory pathways highlights how microtubule stability is coordinately regulated by opposing molecular players after injury.

The activation of STAT signaling via the JAK-STAT pathway in ependymal stem/progenitor cells (epSPCi) enhances neurofilament organization and intermediate filament dynamics by promoting cytoskeletal stabilization and axonal guidance [238]. The FGF-JAK-STAT axis represents a promising but underexplored frontier in axon guidance. While current data suggest a compelling link to cytoskeletal dynamics, critical gaps are rooted in extrapolation from non-neuronal systems and the non-FGF mediated JAK-STAT signaling pathway.

### 4.3. PLCγ Pathway

The phospholipase C gamma (PLCγ) pathway is a critical downstream effector of FGFR signaling. It has been reported that PLCγ emerges as a key regulator of axon guidance, influencing axon pathfinding through cytoskeletal remodeling. In retinal ganglion cells, FGF-2 acts as a chemorepellent that guides the asymmetrical extension of the growth cone only via the PLCγ signaling pathway [21]. The FGF-2-mediated chemorepulsion effect on axon guidance via PLCγ demonstrates the significance of the PLC-γ signaling pathway in FGF-mediated axon guidance. Cytoskeleton dynamics, involving actin filaments, microtubules, and intermediate filaments, underlie growth cone motility. Therefore, in this section, we synthesize the FGF-mediated axon chemorepulsion mechanism from the view of regulation of the cytoskeleton via the PLCγ signaling pathway.

#### 4.3.1. PLCγ Signaling Pathway by FGF Phosphorylation

Upon FGF binding, FGFRs dimerize and undergo autophosphorylation, creating docking sites for adaptor proteins. PLCγ is recruited to activate FGFR via its SH2 domains, where it is phosphorylated and activated [3]. Activated PLCγ hydrolyzes phosphatidylinositol 4,5-bisphosphate (PIP2) into two secondary messengers: inositol 1,4,5-trisphosphate (IP3) and diacylglycerol (DAG) [3]. IP3 binds to IP3 receptors (IP3Rs) on the endoplasmic reticulum, triggering calcium ion (Ca^2^⁺) release into the cytosol from intracellular stores [239,240]. In addition, the elevation of Ca^2^⁺ concentration triggers Ca^2^⁺-induced Ca^2^⁺ release (CICR), which is the other main mechanism for the release of Ca^2^⁺ [241]. DAG activates protein kinase C (PKC), which phosphorylates downstream targets to modulate cellular responses [3]. Grb14, a member of the Grb7 family of adaptors, inactivates PLCγ through reorganization of the PLCγ binding site on the FGFR pY766 residue [3,242].

Surprisingly, intracellular Ca^2^⁺ plays a bifunctional role in axon guidance, mediating both attractive and repulsive responses [239]. Akiyama et al. uncovered the bi-directional turning of growth cone pathfinding with a spatially asymmetric gradient of intracellular Ca^2^⁺ concentration ([Ca^2^⁺]). This reveals the attractive role of a higher-level [Ca^2^⁺] gradient and the repulsive role of a lower-level [Ca^2^⁺] gradient depending on β1-integrin asymmetrical distribution [243]. This supports the assumption of FGF-mediated axon guidance in this review.

#### 4.3.2. IP3 Signaling and Cytoskeletal Remodeling

IP3 signaling plays a multifaceted role in cytoskeletal remodeling during axon guidance, with calcium acting as a pivotal mediator. Hutchins et al., using GnRH neurons, discovered that actin filament dynamics via the regulation of IP3 induced calcium release. After receiving signals from IP3 receptors, Ca^2^⁺ is released and activates the calcium sensor calcium/calmodulin-dependent kinase kinase (CaMKK). CaMKK subsequently activates AMP-activated kinase (AMPK), influencing RhoA/ROCK signaling [244]. IP3, which stimulates Ca^2^⁺ release, coordinates axophilic migration by activating the CaMKK-AMPK-RhoA/ROCK pathway, regulating actin filaments to mediate nucleokinesis. This emphasizes RhoA’s dominance in calcium-dependent cytoskeletal control. Similarly, emerging studies on frontotemporal dementia have revealed IP3-induced Ca^2^⁺ release influences the activation of GSK3β, a key effector of microtubule regulation [245]. This suggests that IP3 may influence axon guidance not only through actin remodeling but also by modulating microtubule networks. Additionally, IP3-induced Ca^2^⁺ release regulates neurofilament organization by promoting the hyperphosphorylation of medium- and high-molecular-weight neurofilament subunits at their tail domains, potentially altering axonal structure and transport [246].

#### 4.3.3. DAG Signaling and Cytoskeletal Remodeling

**Figure 8:**
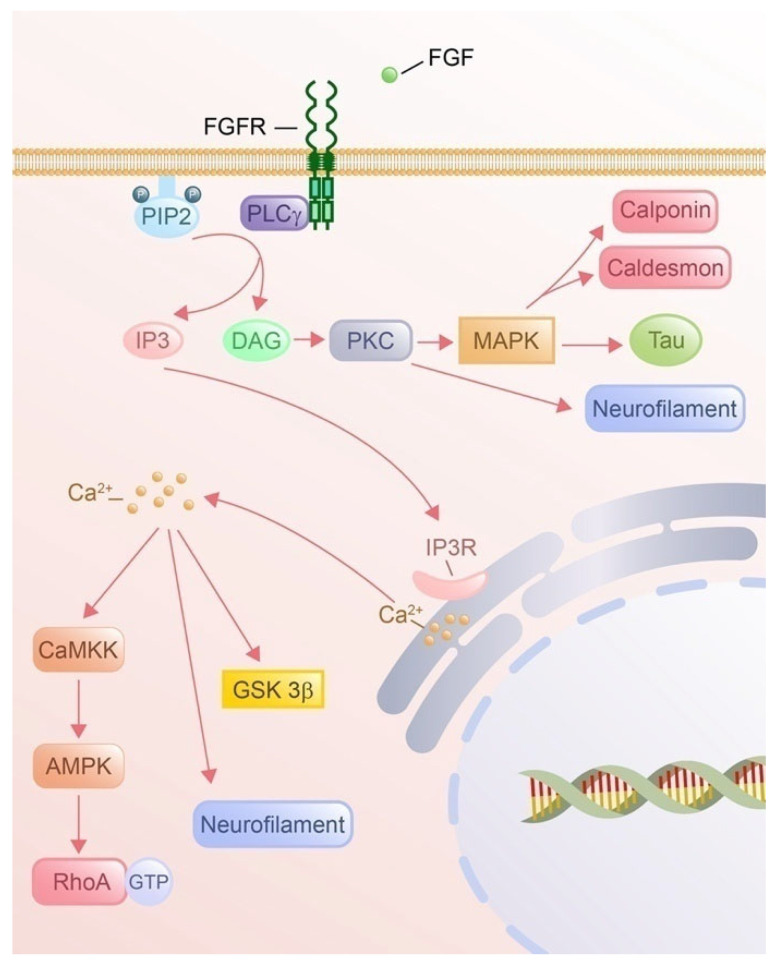
Schematic representation of the PLCγ pathway’s role in cytoskeletal regulation during axon guidance. Activated by FGF signaling, PLCγ hydrolyzes PIP2 to generate DAG and IP3. DAG activates PKC, which phosphorylates actin-binding proteins (calponin and caldesmon) via MAPK to modulate actin polymerization and stress fibers, while PKC-MAPK also targets tau to influence microtubule stability. DAG directly regulates neurofilament phosphorylation, which is critical for axonal integrity. IP3 elevates Ca^2^⁺ levels via IP3 receptors, engaging Ca^2^⁺-dependent kinases like GSK3β and AMPK, which further modulate cytoskeletal dynamics. RhoA GTPase is involved in coordinating actin remodeling. Red arrows: the sequence of signals activation.

DAG signaling regulates cytoskeletal dynamics via PKC-dependent mechanisms (Figure 8). DAG-activated PKC phosphorylates actin-binding proteins, such as calponin and caldesmon, potentially via downstream mitogen-activated protein kinase (MAPK) signaling to modulate actin polymerization and stress fiber formation [247]. PKC also phosphorylates tau, a microtubule-associated protein, via the MAPK pathway, thereby influencing microtubule stability and growth cone dynamics [248]. Beyond its role in PKC activation, DAG directly contributes to cytoskeletal organization by regulating the phosphorylation of low-molecular-weight neurofilament subunits, which are critical for maintaining axonal structure and transport [246]. These findings indicate that DAG, as a significant effector, might through activation of PKC, coordinate actin, microtubule, and neurofilament remodeling via various associated regulatory proteins during axon guidance.

### 4.4. RAS-MAPK Pathway

The RAS-MAPK signaling pathway serves as a critical downstream effector of Receptor Tyrosine Kinases (RTKs), playing a pivotal role in regulating essential cellular processes, such as proliferation, differentiation, survival, and apoptosis. Given the lack of specific experiments, there is no direct evidence to confirm the role of the RAS-MAPK signaling pathway in axon guidance. However, it has been reported that Ras GTPase-activating protein (GAP) p120RasGAP inhibits Ras-MAPK/ERK activation and mediates neurite retraction and growth cone collapse in response to repulsive guidance cues [249]. In addition, brain-derived neurotrophic factor (BDNF)promotes the phosphorylation of ERK and nuclear ERK entry, inducing axonal chemorepulsion [250]. In addition, EphB2, a receptor tyrosine kinase, inhibits the Ras-MAPK pathway, which contributes to cytoskeletal reorganization, promoting growth cone collapse and neurite retraction [251]. These studies indicate that RTK-mediated signals via the Ras-MAPK/ERK signaling pathway regulate cytoskeleton dynamics, ensuring precise navigation of axons toward their targets.

#### 4.4.1. Activation of RAS-MAPK by FGF Signaling

FGF ligands bind to receptor tyrosine kinases (FGFRs), triggering dimerization and autophosphorylation. FGFRs recruit FRS2α as a primary adaptor, binding FGFR via its phosphotyrosine-binding (PTB) domain. Phosphorylated FRS2α recruits GRB2 via its SH2 domain. GRB2 subsequently binds the guanine nucleotide exchange factor (GEF) Son of Sevenless (SOS) via SH3 domains to form the FRS2α-GRB2-SOS complex. This cascade of complex formation catalyzes GDP-to-GTP exchange on RAS, converting it to its active GTP-bound state. This occurs at the plasma membrane where RAS is anchored. Active RAS binds to and activates RAF kinases, including the three isoforms ARAF, BRAF, and CRAF. RAF phosphorylates MAPK/ERK Kinase (MEK) at serine residues. MEK, a dual-specificity kinase, phosphorylates ERK (Extracellular Signal-Regulated Kinase) on threonine and tyrosine residues within its activation loop. Activated ERK (MAPK) is translocated to the nucleus to regulate transcription factors or target cytoplasmic substrates to exert various cellular processes [3].

#### 4.4.2. Cytoskeletal Remodeling via RAS-MAPK Pathway

**Figure 9:**
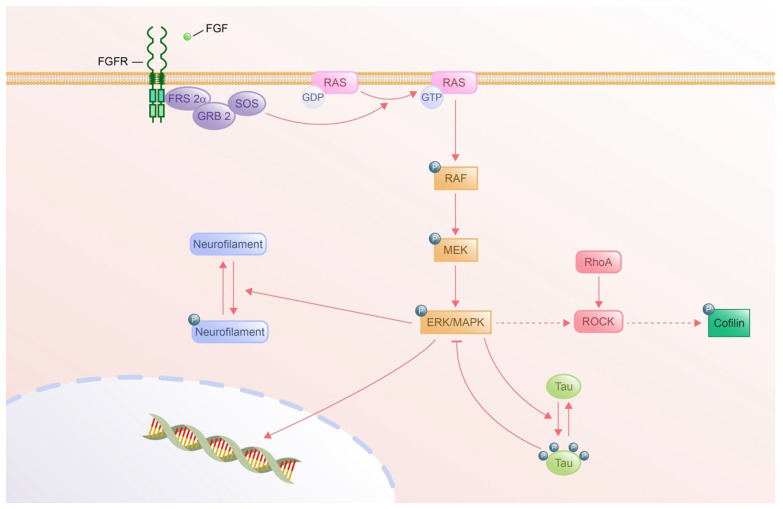
Schematic representation of the RAS-MAPK pathway’s regulation of cytoskeletal dynamics in neurons. The signal passing is driven by FGFR activation via FRS2α and GRB2 to activate RAS-GTP. Downstream, ERK1/2 phosphorylates ROCK, stabilizing actin filaments through cofilin inactivation, while ERK-mediated tau phosphorylation disrupts microtubules; however, tau also exerts negative feedback on ERK to mitigate collapse. This pathway further modulates neurofilament phosphorylation via ERK1/2, promoting neurite retraction. RhoA-ROCK signaling integrates actin stability, while ERK’s dual roles in microtubule destabilization and self-regulation highlight the balance between cytoskeletal remodeling and structural maintenance. Red arrows: the sequence of signals activation; Red dashed arrows: the sequence of signals activation with some steps are omitted here.

The RAS-MAPK pathway is important in the regulation of cytoskeletons, like actin filaments, microtubules, and intermediate filaments, via various associated regulatory proteins in neurons (Figure 9). It has been studied that ROCK is a downstream effector of ERK1/2 in herpes simplex virus 1 entry into neuronal cells. The activation of ROCK induces the phosphorylation of cofilin, promoting actin filament stability [252]. One of the microtubule regulatory proteins, tau, is phosphorylated by phosphorylated ERK in neuroblastoma cells, inducing microtubule disruption during drug therapy [253]. In contrast, a recent study has shown that tau negatively reduces the level of phosphorylated ERK in the retina [254]. The inhibitory effect of tau on phosphorylated ERK effectively intervenes in the microtubule collapse. Moreover, neurite retraction is induced by the phosphorylation of neurofilaments under the control of ERK1/2 [255]. These studies explore the regulation of cytoskeleton dynamics in neurons by the RAS-MAPK signaling pathway.

## 5. Discussion of FGF-Mediated Axon Guidance Mechanism

Although many studies have reported that various FGFs induce axon attraction or axon repulsion, the mechanism of FGF-mediated axon guidance remains unclear. The axon guidance mechanism has been studied so far from two aspects: the cytoskeleton-mediated asymmetric distribution of axon guidance cue receptors and the second messenger switching signals from axon guidance cue to attraction or repulsion. UNC-6/Netrin, an axon guidance cue, triggers axon attraction. The spatial localization of UNC-6/Netrin receptors (UNC-40/DCC) within growth cones is a key determinant of axon guidance. The presence of UNC-6/Netrin mediates the asymmetric distribution of UNC-40/DCC through the cytoskeleton of growth cones in response to extracellular axon guidance cue gradients [11]. However, this model makes it difficult to explain the dual effect of FGF3 and FGF10-mediated axon guidance, which shows axon attraction with high concentrations of FGF3 and FGF10 and axon repulsion with low concentrations of them. In contrast, second messengers, Ca^(2+)^ and cyclic nucleotides mediate the switch of axon attraction and axon repulsion via different signaling pathways triggered by various axon guidance cues [10]. The switch in axon guidance direction under the control of different concentrations is likely to be the mechanism of the dual effect of FGF3 and FGF10.

**Figure 10:**
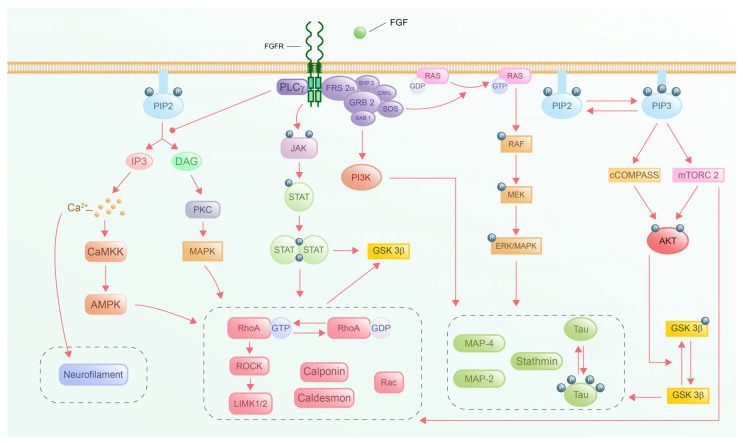
Schematic representation of the FGF downstream multiple signaling pathways. Signaling cascades are initiated by FGF binding to the FGFR on the cell membrane. Key pathways include PLC-γ, which cleaves PIP2 to IP3 and DAG, triggering calcium-related and PKC-MAPK signaling; the JAK-STAT pathway for gene regulation; the Ras-MAPK pathway for protein phosphorylation; and the PI3K-AKT pathway. These pathways interact and regulate downstream cytoskeleton regulatory protein targets, such as Neurofilament, RhoA-related proteins, MAP-4, Tau, etc. Red arrows: the sequence of signals activation.

Axon guidance in response to extracellular cues operates through temporally distinct mechanisms that converge hierarchically on cytoskeletal remodeling. Studies have indicated that FGF plays diverse roles in axon guidance, with its effects varying depending on the neuron type, developmental stage, and local microenvironment [18,19,20,21,22,23,24,25,26,27,28,29,30,31,32,33,34,35,36,37]. This functional variability likely arises from complex interactions among multiple signaling pathways rather than reliance on a single pathway (Figure 10). On the other hand, functional diversity may be attributed to crosstalk between FGF and other guidance pathways (e.g., Netrin, Slit/Robo, Semaphorin) [30,256,257], which is probably exerted by the indirect effects of FGFs on axon guidance. Interestingly, the experiment on the mouse forebrain has provided insights for future studies on the effect of the interaction between the FGF signaling pathway and Slits on axon guidance [257]. In addition, FGF8 has been shown to regulate the expression of semaphorin, which in turn influences the growth of midbrain dopaminergic axons [30]. However, the field lacks abundant evidence to explain how these interactions ultimately converge on cytoskeletal dynamics to steer growth cones. Future research on FGF-mediated axon navigation should prioritize elucidating the specific downstream mechanisms by which these interconnected signaling cascades influence axon guidance and the influence of these interactions.

## 6. Summary and Perspective

FGFs and their receptors play pivotal roles in axon guidance by orchestrating cytoskeletal dynamics through a network of downstream signaling pathways. The interplay between FGF-FGFR complexes, co-factors (heparan sulfate proteoglycans and Klotho proteins), and intracellular cascades—PI3K-Akt, JAK-STAT, PLCγ, and RAS-MAPK—enables the precise regulation of actin filaments, microtubules, and intermediate filaments. Actin dynamics, driven by Rho GTPases, cofilin, profilin, and formins, mediate the growth cone protrusion and retraction. Microtubules, mainly modulated by MAPs and stathmin, provide structural integrity and directional persistence, while intermediate filaments contribute to mechanical resilience and influence axon caliber. Asymmetric activation of these pathways, coupled with spatial gradients of guidance cues, allows the growth cones to navigate complex extracellular environments.

This review synthesizes FGF’s role in cytoskeletal remodeling; however, critical gaps persist. For instance, the mechanism by which FGF3/FGF10 elicits concentration-dependent attraction/repulsion remains hypothetical. We propose that the asymmetric activation of PLCγ or PI3K isoforms underlies this switch, which can be tested via live imaging of second messengers in growth cones. In general, our review mainly focuses on the direct FGF signaling pathways in the cytoskeleton, as the indirect FGF-induced axon guidance mechanism and interaction among these signaling pathways are still not clear. Unresolved questions persist, such as the exact spatiotemporal coordination of signaling crosstalk and the role of intermediate filaments in active guidance. Additionally, the contribution of endocrine FGFs (e.g., FGF21) to axon guidance is virtually unstudied, representing a key frontier in neural development research. Endocrine FGFs may affect axon guidance via activation by other FGF signaling pathways. Future research should focus on unraveling the crosstalk between signaling pathways and their context-specific roles in cytoskeletal remodeling. Advanced techniques may decode how FGF gradients and receptor clustering translate into directional responses. Additionally, intermediate filaments require further exploration to define their contributions to mechano-transduction and axon pathfinding. Addressing these challenges will advance both the fundamental understanding and clinical strategies for neural repair and development.

## Figures and Tables

**Table 1:** Different Effects of FGFs on Neurons in Nervous System.

Classification	Subfamily	FGFs	High Affinity FGFRs [38]	Effects on Neurons
Paracrine (Autocrine)	FGF1 subfamily	FGF1	FGFR1–4	Axon regeneration [22]
		FGF2	FGFR1, 2c, 3c, 4	Axon guidance: attractive/repellent [18,19,20,21]
	FGF4 subfamily	FGF4	FGFR1c, 2c, 3c, 4	Axon guidance: Attractive [18,19]
		FGF5	FGFR1c, 2c, 3c, 4	Regulation [24]
		FGF6	FGFR1c, 2c, 4	Inhibition of axon regeneration [23]
	FGF7 subfamily	FGF3	FGFR1b, 2b	Axon guidance: attractive (low concentration)/repellent (high concentration) [25,27]
		FGF7	FGFR2b	Synapse differentiation [28]
		FGF10	FGFR1b, 2b	Axon guidance: attractive (low concentration)/repellent (high concentration) [26,27] Synapse differentiation [28]
		FGF22	FGFR1b, 2b	Synapse differentiation [28]
	FGF8 subfamily	FGF8	FGFR1c, 2c, 3c, 4	Axon guidance: attractive (directly)/repellent (indirectly) [29,30]
		FGF17	FGFR2c, 3c, 4	Axon navigation (indirectly) [31]
		FGF18	FGFR3c, 4	Increase of neuron number [32,33]
	FGF9 subfamily	FGF9	FGFR2c, 3b, 3c	Axon guidance: Attractive [19]
		FGF16	FGFR2c, 3b	Maturation [33]
		FGF20	FGFR1c, 2b, 2c, 3b, 3c, 4	Axon regeneration [18,34]
Intracrine	FGF11 subfamily	FGF11	-	-
		FGF12	-	-
		FGF13	-	-
		FGF14	-	-
Endocrine	FGF15/19 subfamily	FGF15/19	FGFR1c, 2c, 3c, 4	Axon navigation [35]
		FGF21	FGFR1c, 2, 4	Axon outgrowth [36]
		FGF23	FGFR2c, 4	Axon loss [37]

## Data Availability

The data presented in this study are available in the article.
